# *Lactobacillus plantarum* attenuates glucocorticoid-induced osteoporosis by altering the composition of rat gut microbiota and serum metabolic profile

**DOI:** 10.3389/fimmu.2023.1285442

**Published:** 2024-01-09

**Authors:** Siying Li, Xuebing Han, Naiyuan Liu, Jiang Chang, Gang Liu, Siwang Hu

**Affiliations:** ^1^ The Orthopaedic Center, The First People’s Hospital of Wenling, Wenling Hospital of Wenzhou Medical University, Wenling, Zhejiang, China; ^2^ College of Bioscience and Biotechnology, Hunan Agricultural University, Changsha, Hunan, China

**Keywords:** osteoporosis, glucocorticoids, food active ingredients, dexamethasone, probiotics, gut microbes, differential metabolites, *L. plantarum*

## Abstract

**Introduction:**

Osteoporosis, one of the most common non-communicable human diseases worldwide, is one of the most prevalent disease of the adult skeleton. Glucocorticoid-induced osteoporosis(GIOP) is the foremost form of secondary osteoporosis, extensively researched due to its prevalence.Probiotics constitute a primary bioactive component within numerous foods, offering promise as a potential biological intervention for preventing and treating osteoporosis. This study aimed to evaluate the beneficial effects of the probiotic *Lactobacillus plantarum* on bone health and its underlying mechanisms in a rat model of glucocorticoid dexamethasone-induced osteoporosis, using the osteoporosis treatment drug alendronate as a reference.

**Methods:**

We examined the bone microstructure (Micro-CT and HE staining) and analyzed the gut microbiome and serum metabolome in rats.

**Results and discussion:**

The results revealed that *L. plantarum* treatment significantly restored parameters of bone microstructure, with elevated bone density, increased number and thickness of trabeculae, and decreased Tb.Sp. Gut microbiota sequencing results showed that probiotic treatment increased gut microbial diversity and the ratio of Firmicutes to Bacteroidota decreased. Beneficial bacteria abundance was significantly increased (*Lachnospiraceae*_NK4A136_group, *Ruminococcus*, *UCG_005*, *Romboutsia*, and *Christensenellaceae*_R_7_group), and harmful bacteria abundance was significantly decreased (*Desulfovibrionaceae*). According to the results of serum metabolomics, significant changes in serum metabolites occurred in different groups. These differential metabolites were predominantly enriched within the pathways of Pentose and Glucuronate Interconversions, as well as Propanoate Metabolism. Furthermore, treatment of *L. plantarum* significantly increased serum levels of Pyrazine and gamma-Glutamylcysteine, which were associated with inhibition of osteoclast formation and promoting osteoblast formation. *Lactobacillus plantarum* can protect rats from DEX-induced GIOP by mediating the “gut microbial-bone axis” promoting the production of beneficial bacteria and metabolites. Therefore *L. plantarum* is a potential candidate for the treatment of GIOP.

## 1 Introduction

Osteoporosis, one of the prevalent systemic bone disorders, is delineated by diminished bone mineral density(BMD) and microstructural alterations within the bone tissue (1). The traditional view is that osteoporosis occurs due to endocrine disorders, metabolic disorders, and external mechanical forces leading to disturbances in bone remodeling (2). There are typically two types of osteoporosis: 1) Postmenopausal women and the elderly often suffer from primary osteoporosis 2) Diseases or medications that affect bone metabolism can cause secondary osteoporosis (3).

Glucocorticoid (GC) overconsumption stands as the primary contributor to secondary osteoporosis, and GCs are routinely employed as anti-inflammatory and immunosuppressive agents for managing inflammatory and autoimmune conditions, such as bronchial asthma, acute urticaria, and rheumatoid arthritis (4–8). Regrettably, prolonged misuse of GCs frequently culminates in reduced BMD and substantial bone mass loss, heightening the susceptibility to fragility fractures and exacerbating osteonecrosis (9–12). Dexamethasone (DEX)-induced osteoblast dysfunction and apoptosis are recognized as crucial factors contributing to glucocorticoid-induced osteoporosis (GIOP) (13–15).

However, few clinical drugs are currently available for the treatment of osteoporosis. Initially, selective estrogen receptor modulators (SERMs) were employed for this purpose (16, 17). However, the use of SERMs is associated with elevated risks of uterine cancer, breast cancer, cardiovascular disease, and dementia (18, 19). These adverse effects may constrain the prolonged utilization of SERMs in postmenopausal osteoporosis prevention and management programs. Currently, bisphosphonates (alendronate, zoledronic acid, risedronate, and ibandronate) stand as the most frequently utilized drugs for osteoporosis treatment (20). These agents impede bone resorption by inhibiting osteoclast (OC) production, differentiation, activity, recruitment, and promoting apoptosis. Additionally, they foster osteoblast differentiation to enhance bone formation (21). Clinical studies affirm that bisphosphonate therapy significantly diminishes fracture risk across skeletal sites and enhances patients’ BMD (22). Bisphosphonates can be categorized into non-nitrogen-containing bisphosphonates and nitrogen-containing bisphosphonates based on their chemical structure (23, 24). Notably, the nitrogen-containing bisphosphonate alendronate (ALN), widely employed as the primary therapeutic agent for GIOP, serves as the positive control in this study (25, 26). ALNs, recognized for their anti-catabolic effects and high bone affinity, disrupt farnesyl pyrophosphate synthase in the mevalonate pathway within OCs. This interference regulates posttranslational modifications of key proteins, influencing aspects like cytoskeletal organization and crease boundary formation (27, 28). However, these drugs also have non-specific adverse side effects such as ulcers, gastrointestinal toxicity, nephrotoxicity, hypercalcemia and musculoskeletal pain (22, 29). Therefore, there is an urgent need for new highly effective, specific drugs that have no major adverse effects and are suitable for long-term use for the treatment and prevention of osteoporosis.

Numerous clinical investigations have demonstrated the significant impact of the gut microbiome on bone mass, integrity, and overall strength. Both foundational and applied research have highlighted the potential involvement of the gut microbiome in modulating bone metabolism via the intricate gut-brain axis, a regulator of the immune and endocrine systems (30–32). The comprehensive comprehension of intestinal microorganisms has unveiled a clear understanding of the mechanism underlying the interaction between probiotics and the host flora. Probiotics, denoting health-beneficial active microorganisms, exhibit the capacity to stimulate carbohydrate bacterial activity, augment organic acid secretion, ameliorate the intestinal microenvironment’s acidity, enhance intestinal antimicrobial prowess, and restrain the proliferation of pathogenic bacteria (33). Thus, they are crucial in maintaining intestinal function and equilibrium within the gut microbiota. Notably, substantial research has focused on investigating the potential of probiotics to mitigate osteoporosis in recent years. Within this context, Lactobacillus spp. have emerged as significant contenders (34, 35).

*Lactobacillus plantarum* (*L. plantarum*) is a vital member of the genus *Lactobacillus* and is commonly found in many fermented foods, such as dairy products like yogurt, sauces, fermented cooked meat products and pickles, which are important active ingredients in many foods (36–38). Marina Morato-Martínez et al. completed a 78-person clinical trial showing that patients at risk for osteoporosis (n = 39) adhered to a 24-week regimen of yogurt containing *Lactobacillus plantarum 3547* with a significant increase in bone mass and BMD (39). While several studies in recent years have highlighted the mitigating effect of *L. plantarum* on ovariectomy-induced osteoporosis (40–43), there is a notable gap in research confirming its palliative impact on GIOP and reduction of bone loss.

This study aimed to investigate the therapeutic potential of *L. plantarum* for GIOP. *L. plantarum* was administered by gavage to glucocorticoid dexamethasone-induced osteoporotic rats, and BMD and bone tissue structure were examined. To determine the therapeutic effect of *L. plantarum* on GIOP. High-throughput sequencing of gut contents and serum metabolomics were employed to analyze changes in gut microbiota and metabolic profiles *in vivo*. Also, alendronate treatment was compared with *L. plantarum* treatment. To provide a new theoretical basis and explore the mechanism of *L. plantarum* as a drug candidate for GIOP.

## 2 Materials and methods

### 2.1 Culture of *Lactobacillus plantarum*

*Lactobacillus plantarum* KRHPS1 (*L. plantarum*) was isolated from pickles in Hunan, China, and stored in 30% glycerol test tubes at -80°C in the Microbiology Laboratory, Hunan Agricultural University (Changsha, China). *L. plantarum* was incubated in deMan, Rogosa, and Sharpe agar at 37°C for 18 hours (MRS; Solarbio Science & Technology Co., Ltd., Beijing, China). Following centrifugation (6,000 g, 10 min, 4°C) to eliminate the supernatant, the bacteria were collected from MRS broth cultures. The resulting pellet underwent two washes with phosphate buffer solution (PBS). The viable bacteria were subsequently suspended in sterile saline. The administered dose of the bacterial suspension for gavage was adjusted to 1.0 × 10^9^ CFU/day/rat.

### 2.2 Animal experimental design and treatment

An animal experiment was conducted in adherence to the guidelines for laboratory animal care and utilization set forth by Hunan Agricultural University. Approval was obtained from the Animal Care Committee of Hunan Agricultural University (2022090). 12-week-old SPF SD female rats were obtained from SLAC Laboratory Animal Center (Changsha, China). Dexamethasone was purchased from Suicheng Pharmaceutical, Co., Ltd. (Henan, China). Alendronate was purchased from ovi Pharma Industrial Services, S.A. (SPAIN).

**Figure 1:**
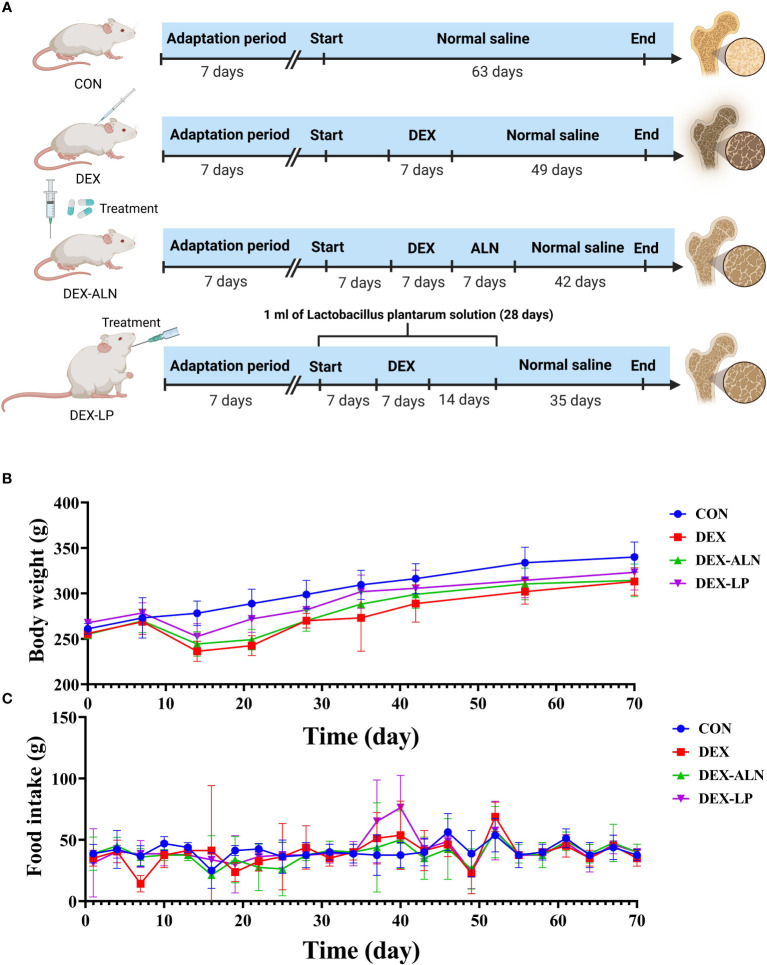
Experimental Design Overview. **(A)** Experimental Group Allocation and Workflow: Twenty rats were allocated into four distinct groups (CON, DEX, DEX-ALN, and DEX-LP), spanning a 70-day experimental duration. **(B)** Body Weight Fluctuations within the Four Rat Groups (n = 5). **(C)** Variation in Food Consumption across Experimental Groups.

All rats were housed in pathogen-free chambers with free access to food and water (temperature, 24 ± 2°C; humidity, 50 ± 5%; light/dark cycle, 12 h). After 7 days of acclimatization, the rats were randomly divided into 4 groups (n = 5/group): (1) Basic diet group (CON); (2) Glucocorticoid dexamethasone-induced osteoporosis group (DEX); (3) Alendronate-treated osteoporosis group (DEX-ALN); and (4) *L. plantarum*-treated osteoporosis group (DEX-LP). Starting on day 7, all rats in the DEX-LP group received 1 ml of *L. plantarum* solution (1.0 × 109 CFU/mL) by gavage daily for four weeks. The CON, DEX, and DEX-ALN groups received equal saline (1 ml) by oral gavage daily. From day 14, the DEX, DEX-ALN, and DEX-LP groups were injected with the glucocorticoid dexamethasone (2.5 mg/kg/d) for one week (44–47). From day 21, the DEX-ALN group received alendronate (1.517 mg/kg) by oral gavage for one week (44). From the sixth week, no disturbance was done to the rats until the tenth week of sampling. During the experimental period, rat body weights were assessed weekly, while water and food intake were quantified every three days. On the final day of the tenth week, rats underwent a 12-hour fast, although they retained ad libitum access to water. Ultimately, euthanasia was performed to conclude the experimental protocol (**Figure 1A**).

### 2.3 Sample collection

Sampling involved the intraperitoneal administration of 5% pentobarbital sodium for rat anesthesia, followed by the collection of blood samples through cardiac puncture. Serum was isolated through centrifugation at 4° and 4000g for 10 minutes, subsequently stored at -80°C in preparation for metabolomics analysis. The rats were then cervically dislocated and killed. The 1-2 cm colonic contents were collected and stored in EP tubes labeled and labeled. Immediately transferred to -80°C refrigerator for gut microbial 16S rRNA analysis. The right femur of the rat was dissected and isolated from the posterior part of the right femur, and the attached muscles and tendons were removed to obtain the right leg femur of the rat fixed in paraformaldehyde for and Haematoxylin and eosin (HE) staining analysis.

### 2.4 Micro-computed tomography (Micro-CT) and HE staining of rat femurs

Femur samples from rats were subjected to scanning using the Bruker Micro-CT Skyscan 1276 system (Kontich, Belgium). Subsequent 3D and 2D analyses were performed using CT Analyser software (version 1.20.3.0). The parameters were adjusted and then calculated to obtain: Trabecular separation (Tb.Sp), Trabecular number (Tb. N), Bone volume (BV) and Tissue volume (TV).

The rat femur after Micro-CT scanning was decalcified with 10% EDTA and embedded in paraffin. Sections were made and stained according to standard instructions.

### 2.5 16S ribosomal RNA amplicon sequencing

Colon contents from rats were combined with ground beads and lysate. The mixture was agitated and then subjected to centrifugation to eliminate the supernatant. Microbial DNA within the colon contents was extracted using the QIAamp DNA Stool Mini Kit (QIAGEN, Hilden, Germany), following the instructions. Subsequently, equimolar mixing was conducted for library construction, followed by sequencing on a MiSeq platform (PE300). Offline data analysis was carried out, with assessment of α diversity conducted using mothur (Version 1.33.3). To visualize inter-group species variations, species composition maps were generated at the phylum, family, genus, and species levels.

### 2.6 Serum metabolomic analyses

We thawed 100 μL serum samples stored at -80°C and supplemented them with 300 μL of methanol (Merck, Darmstadt, Germany) extract. Subsequently, 10 μL of DL-o-chlorophenylalanine (Sigma, St. Louis, MO) was added and thoroughly mixed. The resultant samples underwent centrifugation at 12000 rpm and 4°C for 15 minutes. The resulting supernatant was carefully transferred to a liquid-phase vial for subsequent analysis. Each sample underwent a liquid chromatography-mass spectrometry (LC-MS) analysis platform equipped with a Hyper gold C18 column (3 μm, 100 × 4.6 mm) from Waters, Dublin, Ireland. Specific parameter configurations and data analysis were carried out in alignment with previously established protocols (48).

### 2.7 Statistical analysis

We evaluated variance homogeneity through Levene’s test and subsequently applied Student’s t-test, utilizing one-way analysis of variance (ANOVA). These analyses were executed utilizing IBM SPSS Statistics 21 for Windows. For the exploration of potential associations between intestinal microbes and serum metabolites, we employed GraphPad Prism 7. Correlations between colonic microorganisms, serum metabolites, and femoral parameters were evaluated by Pearson’s correlation coefficient. Statistical significance was designated with a *P*-value below 0.05.

## 3 Results

### 3.1 Effect of *L. plantarum* on dexamethasone-induced osteoporosis in rats

Before the dexamethasone injection at week 3, all groups gained weight, and after the third week all lost weight. However, alendronate and *L. plantarum* seemed to slow down this decline. After stopping the dexamethasone injection, all three groups gradually gained weight, with the DEX-ALN and DEX-LP groups showing closer weight to the CON group compared to the DEX group (**Figure 1B**). Furthermore, an assessment of food intake was conducted within each rat group. Remarkably, no substantial differences in food consumption emerged among the groups, even within the DEX group characterized by GIOP (**Figure 1C**).

### 3.2 *L. plantarum* alleviates dexamethasone-induced osteoporosis

**Figure 2:**
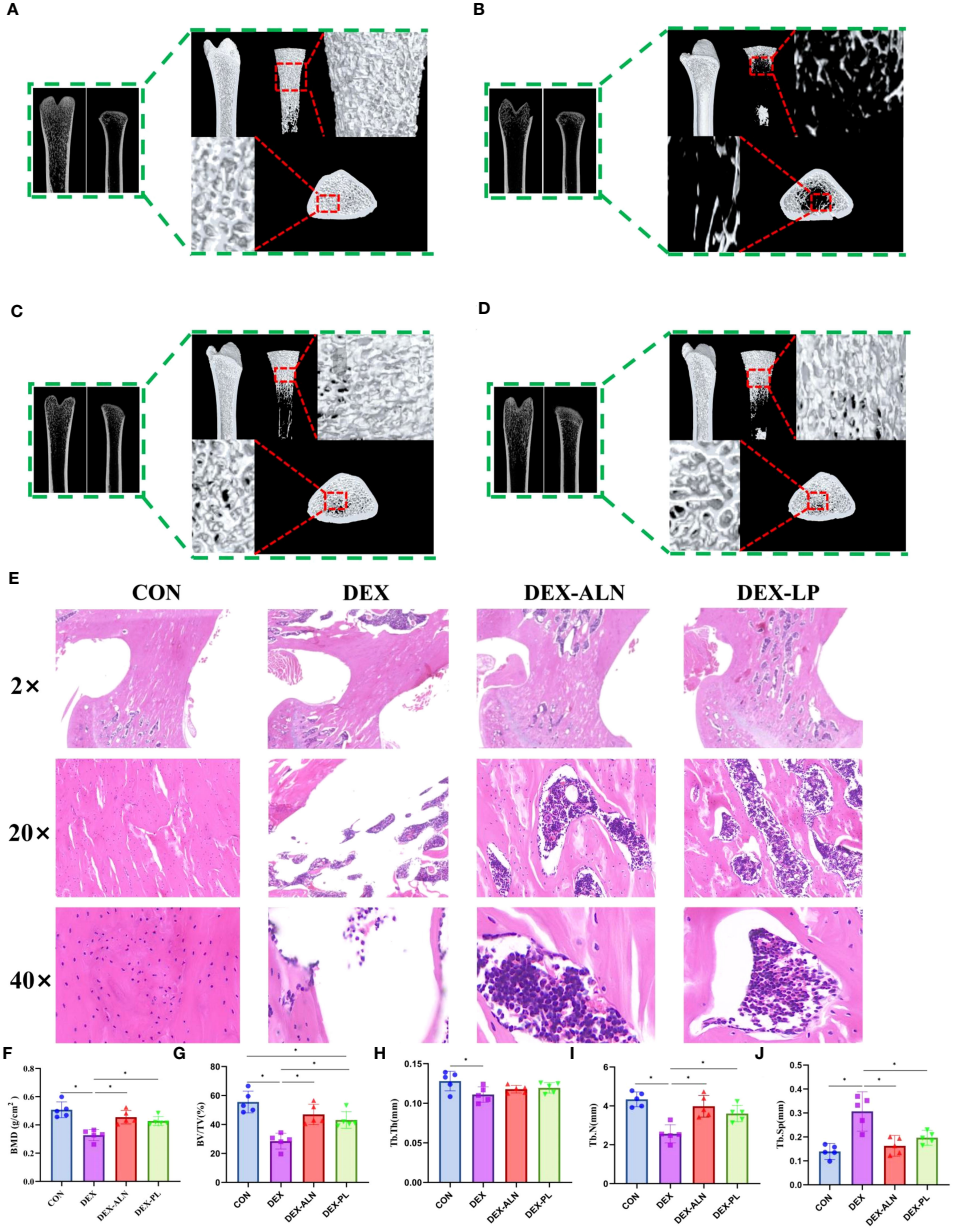
*L. plantarum* showed therapeutic effects on glucocorticoid dexamethasone-induced osteoporosis similar to the therapeutic drug alendronate. **(A–D)** Micro-CT scans of the femur, illustrating representative samples from each rat group, encompass both 2D reconstructions of the proximal and distal femur, along with a 3D reconstruction of the proximal femur; **(E)** Representative HE-stained images of the proximal femur in each group, with magnifications including 2x, 20x, and 40x; **(F)** Bone mineral density (BMD) of rats in different group; **(G)** Percent bone volume (BV/TV); **(H)** Trabecular thickness (Tb.Th); **(I)** Trabecular number (Tb.N); **(J)** Trabecular separation (Tb.Sp);* *P* < 0.05 (n = 5).

Microcomputed tomography obtained two-dimensional longitudinal and three-dimensional transverse and longitudinal views of the rat’s femur (**Figures 2A**–**D**). Micro-CT scans revealed pronounced deterioration of bone microstructure in the DEX group in comparison to the CON group. The most intuitive manifestation is that the number of bone trabeculae becomes less, the thickness becomes thinner, the volume becomes smaller, the bone trabeculae are obviously separated, and the bone marrow cavity becomes larger. The bone cortex and bone trabeculae microstructure was observed by further HE staining of rat femur. In contrast to the CON group, the DEX group exhibited reduced cortical bone thickness, accompanied by corresponding thinning and decreased density of the trabecular structure (**Figure 2E**). This is confirmed by comparing the parameters in **Figures 2F**–**J**. BMD, BV/TV, Tb.Th, Tb.N significantly decreased and Tb.Sp significantly increased after dexamethasone treatment (P<0.05), all five parameters indicating significant bone loss. Collectively, these findings affirm the successful establishment of the rat model for GIOP.

Compared with the DEX group, the DEX-ALN and DEX-PL groups showed a significant increase in BMD, BV/TV, and Tb.N (P< 0.05) and a significant decrease in Tb.Sp (P < 0.05). Micro-CT images further confirmed the reversal of femoral microstructural damage in GIOP rats by alendronate and *L. plantarum*. The structure of the bone trabeculae is restored and the marrow cavity is minimized. HE staining showed that cortical bone thickness was restored in the DEX-ALN and DEX-LP groups. By all indicators, the potential of *L. plantarum* for the treatment of GIOP is comparable to alendronate.

### 3.3 *L. plantarum* regulates gut microbes in dexamethasone-induced osteoporotic rats

The gut microbiota maintains a significant connection with osteoporosis. To ascertain the influence of *L. plantarum* on the gut microbiota, we examined 16S rRNA sequences from the v3-v4 region, extracted from colonic samples of four rat groups. Sequencing yielded a total of 1,158,576 read pairs across 20 samples, which were subsequently processed to generate 1,091,136 high-quality clean reads following double-ended read quality control and merging. Each sample provided a minimum of 37,941 clean reads, with an average of 58,194 clean reads.

#### 3.3.1 *L. plantarum* modulates the alpha diversity in the colon of dexamethasone-induced osteoporotic rats

**Figure 3:**
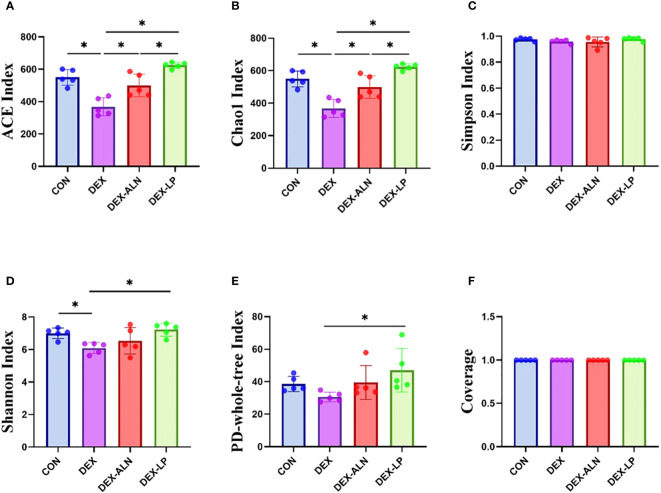
Microbial alpha diversity analysis of colonic contents between four groups of rats. **(A)** ACE index, **(B)** Chao 1 index, **(C)** Simpson index, **(D)** Shannon index, **(E)** PD-whole-tree index and **(F)** Coverage. data are mean ± SD, analyzed by one-way ANOVA. *P < 0.05 (n = 5).

The α-diversity results indicated that the diversity of the colonic microbiota was reduced in the rat model of dexamethasone-induced osteoporosis (**Figure 3**). Treatment with alendronate or *Lactobacillus plantarum* reversed this trend. The ACE, Chao 1, Shannon, and PD-whole-tree indices were significantly higher in the DEX-LP group compared with the DEX group, and even higher than the CON group. Thus, administration of *Lactobacillus plantarum* greatly counteracted the GIOP-induced reduction in gut microbial diversity.

#### 3.3.2 *L. plantarum* affects the abundance of phylum-level microorganisms during glucocorticoid-induced osteoporosis in rats

**Figure 4:**
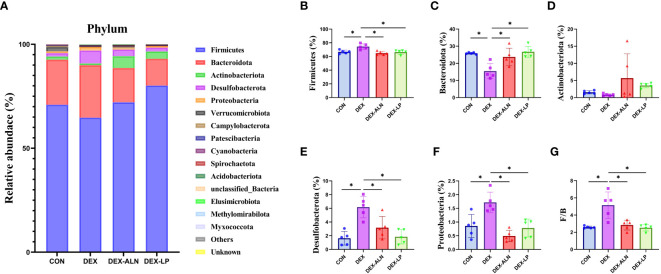
Microbial composition analyzed at the phylum level. **(A)** Relative abundance of microbial gates in rat colon. Comparison of the relative abundance of Firmicutes **(B)**, Bacteroidota **(C)**, Actinobacteriota **(D)**, Desulfobacterota **(E)**, and Proteobacteria **(F)** in the colons of rat in the CON, DEX, DEX-ALN, and DEX-LP groups, and the F/B value **(G)**. *P < 0.05 (n = 5).

Changes in the composition and proportions of intestinal microorganisms are closely related to the pathogenesis of GIOP, and we analyzed the sequencing results in a specific taxonomic manner so as to determine the composition and structure of the flora of the rat colon contents at different taxonomic levels. At the phylum level, Firmicutes, Bacteroidota, Actinobacteriota, Desulfobacteriota, and Proteobacteriota had the highest abundance, accounting for more than 99% of all microorganisms (**Figure 4A**). In contrast to the CON group, the DEX group exhibited notably elevated abundance levels of Firmicutes, Desulfobacteriota, and Proteobacteriota (*P* < 0.05, **Figures 4B, E, F**). Moreover, the abundance of Bacteroidota decreased by 10.38% (*P* < 0.05, **Figure 4C**), leading to an increased F/B ratio (*P* < 0.05, **Figure 4G**). Alendronate and *L. plantarum* can reverse this trend. Firmicutes, Desulfobacteriota, Proteobacteriota were significantly decreased in DEX-LP and DEX-ALN groups compared to DEX group (*P* < 0.05, **Figures 4B, E, F**). In this aspect, *L. plantarum* seems to be more capable than alendronate. In addition, the reldeative abundance of Actinobacteriota was 5.69% in the DEX-ALN group and 3.53% in the DEX-PL group, while it was only 0.85% in the DEX group and 1.6% in the CON group. Treatment with alendronate or *L. plantarum* increased the abundance of Actinobacteriota (**Figure 4D**). After probiotic treatment, Bacteroidota abundance was significantly increased compared to the DEX group (**Figure 4C**). The F/B reached 2.74 and 2.48 in the DEX-LP and DEX-ALN groups. The DEX-LP group’s F/B is even lower than the CON group’s 2.58 (**Figure 4G**).

#### 3.3.3 *L. plantarum* affects the abundance of order microorganisms during glucocorticoid-induced osteoporosis in rats

**Figure 5:**
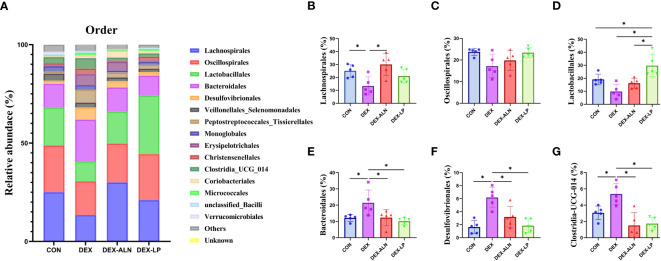
Microbial composition analyzed at the order level. **(A)** Relative abundance of microbial gates in rat colon. Comparison of the relative abundance of Lachnospirales **(B)**, Oscillospirales **(C)**, Lactobacillales **(D)**, Bacteroidales **(E)**, Desulfovibrionales **(F)**, and Clostridia_UCG_014 **(G)** in the colons of rat in the CON, DEX, DEX-ALN, and DEX-LP groups. *P < 0.05 (n = 5).

The levels of the top 15 bacterial orders in the rat colon contents are shown in **Figure 5A**. The top four microorganisms included Lachnospirales, Oscillospirales, Lactobacillales, and Bacteroidalies, which accounted for more than 70%. Compared to the CON group, DEX treatment reduced the abundance of Lachnospirales, Oscillospirales, and Lactobacillales by 11.7%, 6.6%, and 9.3%, respectively (**Figures 5B–D**). In addition, the abundance of Bacteroidales, Desulfovibrionales and Clostridia_UCG_014 was significantly elevated (*P* < 0.05, **Figures 5E–G**). The abundance of Lachnospirales (30.0%), Bacteroidales (12.3%), Desulfovibrionales (3.2%) and Clostridia_UCG_014 (1.5%) was restored after treatment with sodium alenphosphate (**Figures 5B, E–G**). Abundance changes in Oscillospirales and Lactobacillales showed an increasing trend, although not statistically different (**Figures 5C, D**). *L. plantarum* gavage showed similar effects to alendronate, both reversing microbial abundance alterations caused by dexamethasone injection. Abundance of Lactobacillales (29.7%), Bacteroidales (10.1%), Desulfovibrionales (1.8%), and Clostridia_UCG_014 (1.7%) was significantly restored (*P* < 0.05, **Figures 5D–G**).

#### 3.3.4 *L. plantarum* affects the abundance of genus-level microorganisms during glucocorticoid-induced osteoporosis in rats

**Figure 6:**
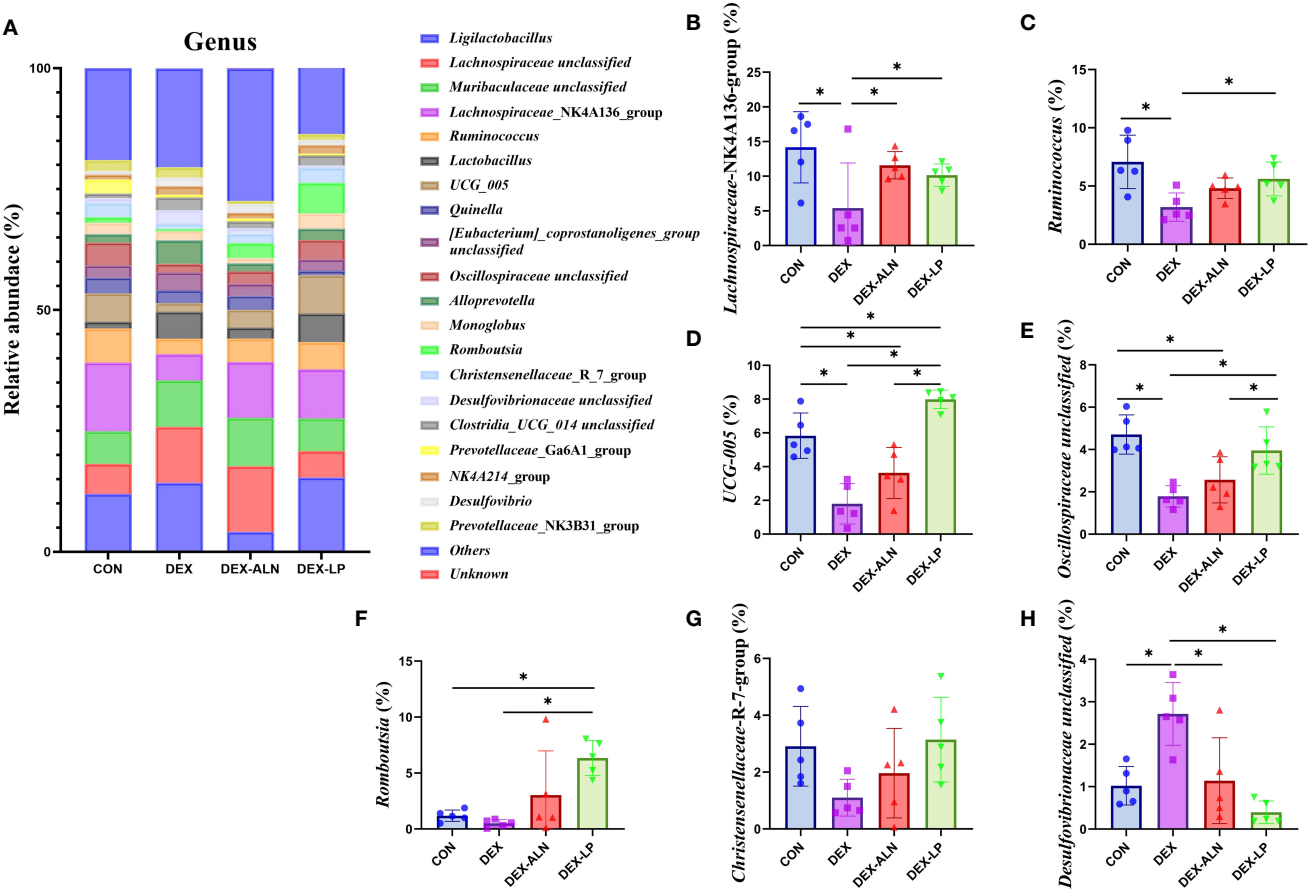
Microbial composition analyzed at the genus level. **(A)** Relative abundance of microbial gates in rat colon. Comparison of the relative abundance of *Lachnospiraceae*_NK4A136_group **(B)**, *Ruminococcus* **(C)**, UCG_005 **(D)**, *Oscillospiraceae* unclassified **(E)**, and *Romboutsia* **(F)**, *Christensenellaceae*_R_7_group **(G)** and *Desulfovibrionaceae* unclassified **(H)** in the colons of rat in the CON, DEX, DEX-ALN, and DEX-LP groups. *P < 0.05 (n = 5).

By analyzing the composition of rat colon microorganisms, we selected the top 15 genera in terms of abundance for analysis (**Figure 6A**). The main genera were *Lgilactobacillus*, *Lachnospiraceae unclassified*, *Muribaculaceae unclassified*, *Lachnospiraceae*_NK4A136_group and *Ruminococcus* etc. In CON, DEX, DEX-ALN and DEX-LP groups, the abundance of *Lachnospiraceae*_NK4A136_group was 14.2%, 5.4%, 11.6% and 10.2%, respectively (**Figure 6B**). The abundance of *Ruminococcus* was 7.1%, 3.2%, 4.8% and 5.6%, respectively (**Figure 6C**). The abundance of UCG_005 was 5.8%, 1.8%, 3.6% and 8.0%, respectively (**Figure 6D**). The abundance of *Oscillospiraceae* unclassified was 4.7%, 1.8%, 2.6% and 4.0%, respectively (**Figure 6E**). The abundance of *Romboutsia* was 1.2%, 0.5%, 3.0% and 6.3%, respectively (**Figure 6F**). *Christensenellaceae*_R_7_group had abundances of 2.9%, 1.1%, 2.0% and 3.1%, respectively (**Figure 6G**). *Desulfovibrionaceae* unclassified had abundances of 1.0%, 2.7%, 1.1% and 0.4%, respectively (**Figure 6H**). Compared to the CON group, the abundance of *Lachnospiraceae*_NK4A136_group, *Ruminococcus*, UCG_005, and *Oscillospiraceae* unclassified decreased significantly in the DEX group, and *Desulfovibrionaceae* unclassified increased significantly. Treatment with alendronate and *L. plantarum* restored these changes (*P* < 0.05).

#### 3.3.5 LEfSe analysis of microbiota in colon content

**Figure 7:**
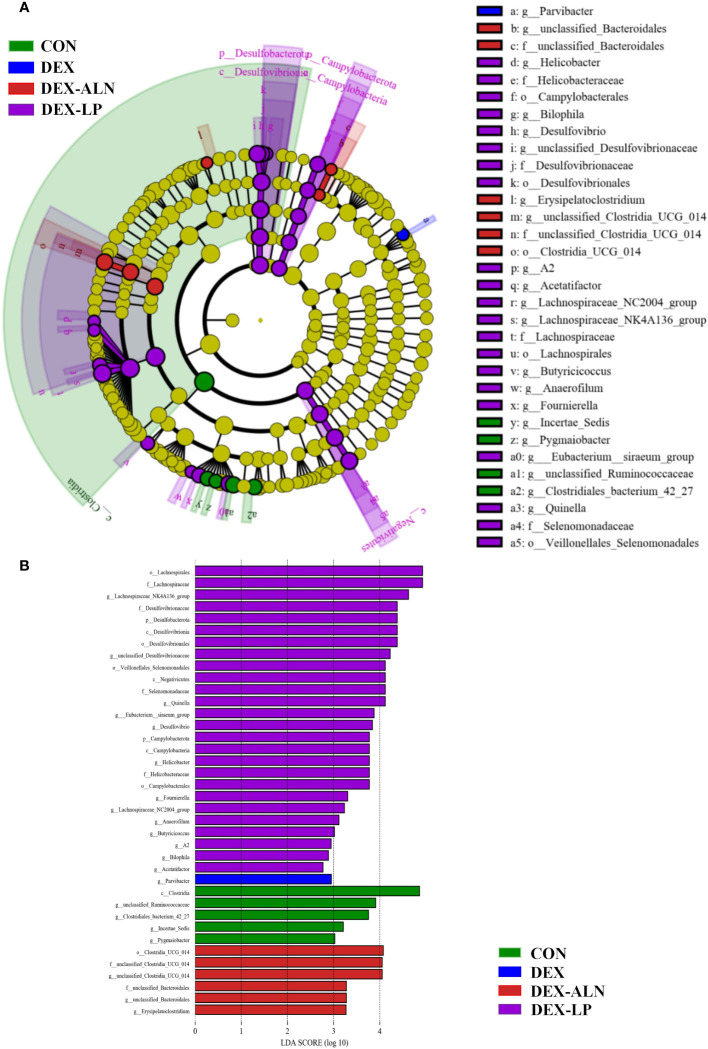
Colonic microorganisms were characterized in GIOP rats by *L. plantarum* gavage and alendronate injection. **(A)** LEfSe taxonomic cladogram, different colors indicate the enrichment of specific taxa in the CON group (green), DEX group (red), DEX-ALN group (blue), and DEX-LP (purple); **(B)** LDA scores, LDA scores higher than 2 were considered to be significant contributors to the model.

LEfSe analysis of colonic microbes in the CON, DEX, DEX-ALN, and DEX-LP groups is shown in **Figure 7A** (*P* < 0.05). The CON group was significantly enriched in *Incertae_Sedis*, *Pygmaiobacter*, *unclassified Ruminococcaceae* and *Clostridiales bacterium*_42_27; The DEX group was significantly enriched in *Parvibacter*; the DEX-ALN group was significantly enriched in unclassified_Bacteroidales, Erysipelatoclostridium, and unclassified_Clostridia_UCG_014; the order Campylobacterales, Veillonellales Selenomonadales,and Desulfovibrionales; family Helicobacteraceae, Desulfovibrionaceae, Lachnospiraceae, andSelenomonadaceae; genus *Desulfovibrio*, *Lachnospiraceae*_NC2004_group, *Lachnospiraceae*_NK4A136_group, *Butyricicoccus*, *Anaerofilum*, and *Quinella* were significantly enriched in the DEX-LP group. Subsequently, Linear Discriminant Analysis (LDA) was employed to gauge the influence of species abundance on inter-group disparities. Species exhibiting distinct abundance across groups were identified by identifying LDA scores surpassing 2 (**Figure 7B**). In DEX-LP group, the species abundance of the phylum Desulfobacterota and Campylobacterota; class Desulfovibrionia, Negativicutes and Campylobacteria; order Desulfovibrionales and Veillonellales_Selenomonadales and Campylobacterales; family Lachnospiraceae, Desulfovibrionaceae, Selenomonadaceae and Helicobacteraceae has the most significant impact on the abundance of differential bacteria.

### 3.4 Effect of *L. plantarum* treatment on plasma total metabolite levels in rats

**Figure 8:**
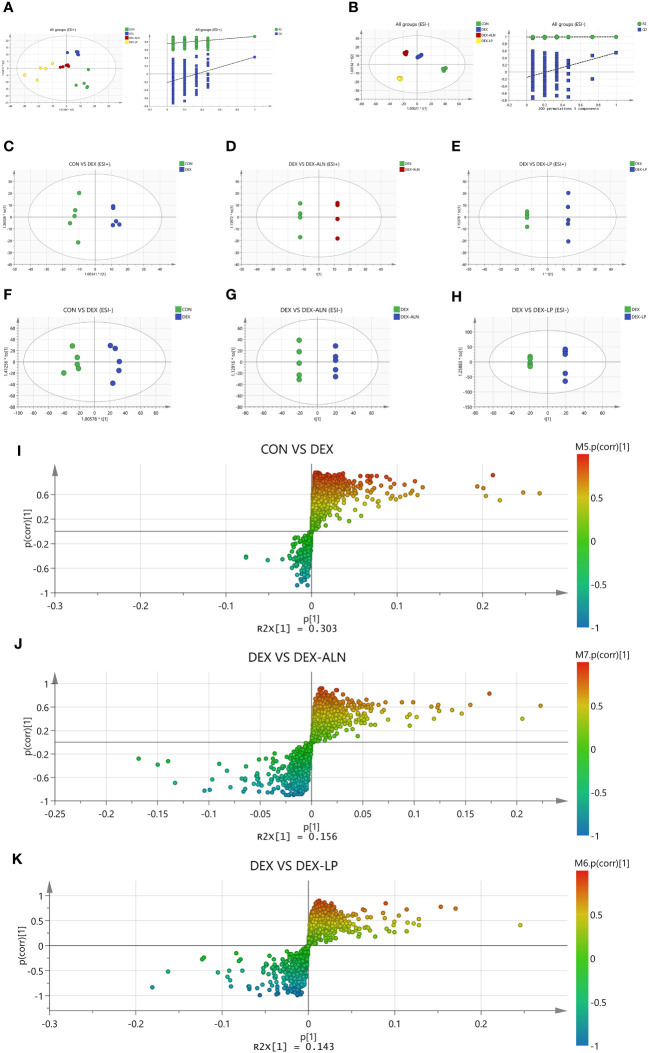
Plots of the multivariate statistical comparisons between groups. **(A)** OPLS-DA score plot of all groups (ESI+) and OPLS-DA (Validate Model) score plot of all groups (ESI+); **(B)** OPLS-DA score plot of all groups (ESI-) and OPLS-DA (Validate Model) score plot of all groups (ESI-); **(C)** OPLS-DA score plot of CON VS DEX (ESI+); **(D)** OPLS-DA score plot of DEX Vs DEX-ALN (ESI+); **(E)** OPLS-DA score plot of DEX Vs DEX-LP (ESI+); **(F)** OPLS-DA score plot of CON VS DEX (ESI-); **(G)** OPLS-DA score plot of DEX Vs DEX-ALN (ESI-); **(H)** OPLS-DA score plot of DEX Vs DEX-LP (ESI-); **(I)** S-plots of serum profiles of CON VS DEX groups scanned by OPLS-DA analysis; **(J)** S-plots of serum curves of DEX VS DEX-ALN groups. **(K)** S-plots of serum curves of DEX VS DEX-LP groups.

Serum metabolites associated with GIOP in rats were quantified using LC-MS. Each scatter plot portrays serum samples from both the positive ion model (**Figure 8A**) and the negative ion model (**Figure 8B**). To establish the connection between metabolite expression and sample classification, orthogonal partial least squares discriminant analysis (OPLS-DA) was employed for predictive modeling of sample categories, followed by model validation. The outcomes revealed substantial variability in serum metabolites across diverse groups, with notable consistency in metabolite profiles within each group.

In order to analyze the variables more accurately, the alteration of overall metabolism in GIOP rats by *L. plantarum* gavage was assessed. OPLS-DA was supervised in positive and negative ion mode by analyzing CON group VS DEX group, DEX group VS DEX-ALN group and DEX group VS DEX-LP group two by two (**Figures 8C–H**). Based on 200 chance permutations, no overfitting was found. The S-plot further shows the differential metabolites in the paired two groups, with p (1) representing the effect of the X variable on the group, and p (corr) (1) representing the reliability of the X variable effect. Thus, the metabolites in the S plot’s lower left and upper right corners are critical (**Figures 8I–K**).

**Figure 9:**
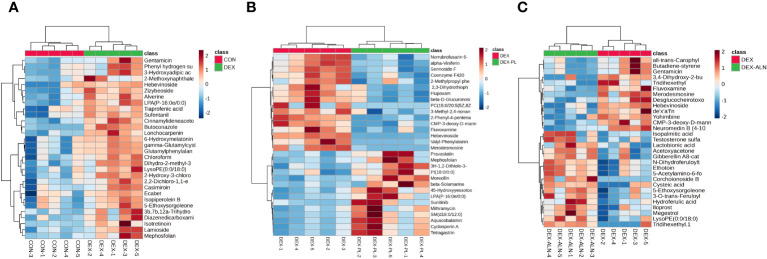
Heat map analysis of differential metabolites. Each column represents a metabolite and each row represents a sample. Red color represents up-regulation of metabolite expression and blue color represents down-regulation of metabolite expression. **(A)** Heat map of CON vs DEX differential metabolites **(B)** Heat map of DEX vs DEX-ALN differential metabolites **(C)** Heat map of DEX vs DEX-LP differential metabolites.

Potential markers were identified through screening based on S-plots, with VIP values exceeding 1 and *P*-values falling below 0.05. Subsequently, the identified differential metabolites were validated using the HMDB and METLIN databases. The top thirty differential metabolites were plotted as heat maps to clearly show their relative amounts and changes (**Figures 9A–C**).

A total of 173 differential metabolites were found by analyzing serum metabolome data from the CON and DEX groups. 87 differential metabolites were found in the DEX and DEX-ALN groups. 90 differential metabolites were found in the DEX and DEX-LP groups. Among them, Fluvoxamine, Hebevinoside XIII, Norrubrofusarin 6-beta-gentiobioside, and 3-Methyl-2,5-furandione were up-regulated in the GIOP modeling group and recovered after *L. plantarum* treatment. In addition, treatment with the GIOP therapeutic drug sodium alenphosphate (DEX-ALN group) significantly reduced increased serum metabolites in the DEX group, including Hebevinoside XIII, Fluvoxamine, Gentamicin, and Arbekacin. However, 3-O-trans-Feruloyleuscaphic acid, LysoPE (0:0/18:0), and LysoPE (0:0/18:0) showed further elevation after alendronate treatment. There were 17 identical differential metabolites in the DEX group VS DEX-LP group and DEX group VS DEX-ALN group. Among them Hebevinoside XIII and Fluvoxamine were elevated in the model group and then decreased after treatment with alendronate or *L. plantarum*.

**Figure 10:**
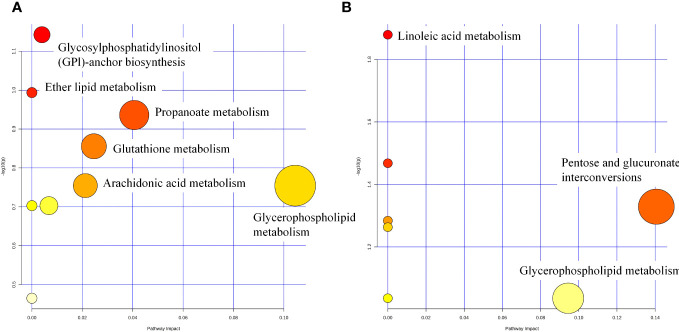
Metabolic pathway analysis of potential biomarkers for CON vs DEX **(A)** and DEX vs DEX-LP **(B)**.

Differential metabolite enrichment analysis showed that between CON and DEX groups, differential metabolites were mainly enriched in Propanoate metabolism (**Figure 10A**), Glutathione metabolism, Arachidonic acid metabolism, and Glycerophospholipid metabolism. Between the DEX and DEX-LP groups, differential metabolites were mainly enriched in Pentose and glucuronate interconversions and Glycerophospholipid metabolism (**Figure 10B**). Differential metabolites that contribute significantly to these pathways include dolichyl D-xylosyl phosphates, Glutamylphenylalanine, gamma-Glutamylcysteine, 6-Hydroxymelatonin, Tetrahydrocorticosterone, 3H-1,2-Dithiole-3-thione and beta-D-Glucuronoside.

### 3.5 Correlations between gut microbes, serum metabolites and femoral parameters

**Figure 11:**
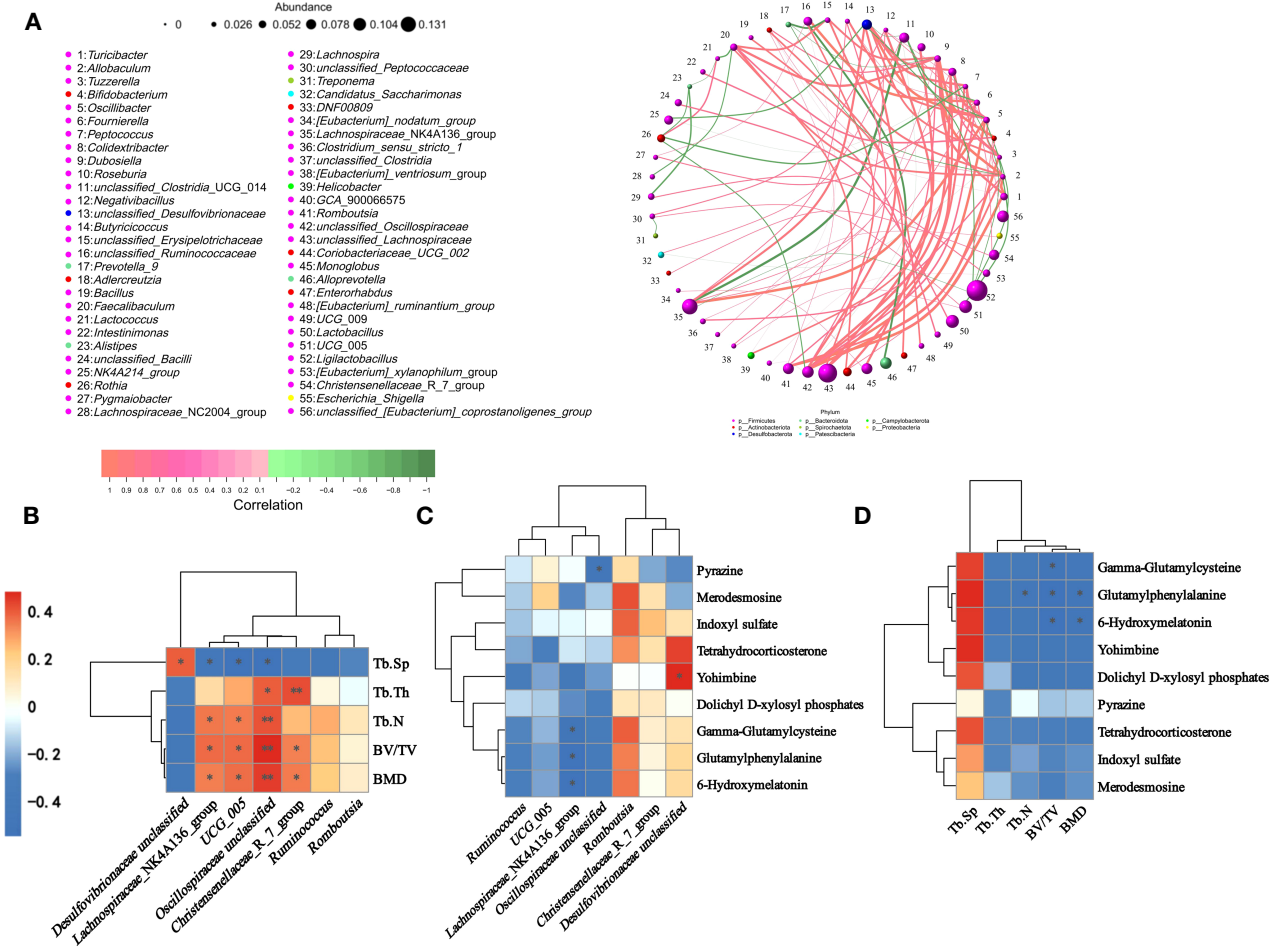
Spearman correlation analysis of gut microbiota, serum metabolites, and femoral parameters. **(A)** Correlation network diagram of gut microbiota in rats. **(B)** Heat map of correlation between gut differential microbiota and femur parameters. **(C)** Heat map showing gut differential microbiota with raw serum differential metabolites. **(D)** Heatmap of correlation between serum differential metabolites and femur parameters. ^∗^ *P* < 0.05 and ^∗∗^ *P* < 0.01.

A genus-level network Spearman correlation analysis of the gut microbiota of each group of rats showed that *Romboutsia*, *unclassified Oscillospiraceae*, and *unclassified Lachnospiraceae* showed a high positive correlation with the other microorganisms, while the *Lachnospiraceae*_NK4A136_group and *unclassified_Clostridia*_UCG_014 showed the strongest negative correlation. *Lachnospiraceae*_NK4A136_group, *unclassified_Lachnospiraceae*, *Lactobacillus*, *UCG*_005 and *Ligilactobacillus* showed the highest relative abundance (**Figure 11A**). Correlations between colonic microbes, serum metabolites and femoral parameters showed that *Lachnospiraceae*_NK4A136_group, *Ruminococcus*, *UCG*_005, *Oscillospiraceae unclassified*, *Romboutsia*, *Christensenellaceae_R*_ 7_group and all differential metabolites shown in the figure were negatively correlated with Tb.Sp, while positively correlated with other parameters (BMD, BV/TV, Tb.Th and Tb.N). In contrast, *Desulfovibrionaceae unclassified* was the opposite (**Figures 11B, D**). The heatmap of correlation between colonic microorganisms and serum differential metabolites showed that *Lachnospiraceae*_NK4A136_group was significantly negatively correlated with Gamma-Glutamylcysteine, Glutamylphenylalanine and 6-Hydroxymelatonin. While *Desulfovibrionaceae unclassified* was significantly correlated positively with Yohimbine (**Figure 11C**).

## 4 Discussion

Osteoporosis, the fourth most common chronic disease, is characterized by decreased bone mass and deterioration of group tissue structure. As the development of osteoporosis is asymptomatic, it is often clinically diagnosed after a fracture, which means that it is difficult to treat effectively at an early stage (49, 50). Therefore, fragility fractures are usually accompanied by a significant and irreversible decline in bone function (51). GIOP is the most common of the drug-induced osteoporosis (52). It causes bone remodeling imbalance by inhibiting osteoblast differentiation; promoting osteoclast (OC) activation and proliferation; and inducing osteoblast apoptosis, which ultimately leads to osteoporosis (53, 54). Unlike osteoporosis caused by increasing age and menopause in women, glucocorticoids cause rapid bone resorption and bone loss early in treatment, with a rapid decline in BMD, followed by a severe long-term suppression of bone formation, with OP occurring in more than 41.4% of patients during long-term treatment with GC (52). Although a variety of drugs have been used to treat GIOP with varying degrees of effectiveness, they can cause a number of potential adverse side effects. Therefore, the discovery of new drugs or alternative strategies has attracted much attention. Since the gastrointestinal tract has a complex role in maintaining bone health through the “gut-bone” axis (55–58). The gut microbiota of patients with bone loss and OP differs from that of healthy individuals, revealing that gut microbes may be a potential target for OP prevention and treatment.

This study investigated the effects and mechanisms of the probiotic *L. plantarum* on GIOP in rats. The study results showed that *L. plantarum* possessed similar therapeutic and preventive effects to alendronate (a common treatment for osteoporosis) as seen in the Micro-CT, gut microbiome, and serum metabolome. Micro-CT results showed that *L. plantarum* restored the damaged bone microstructure, with a significant increase in trabecular parameters (mineral density, bone volume fraction, number of trabeculae, and thickness of trabeculae) and a reduction in the marrow cavity. Sequencing results of colon microorganisms showed that *L. plantarum* significantly regulated the structure of rat gut microbial community, with a significant increase in F/B. In addition, the abundance of beneficial bacteria *Lachnospiraceae*_NK4A136_group, *Ruminococcus*, *UCG_005*, and *Romboutsia* was significantly increased and the abundance of harmful bacteria *Desulfovibrionaceae* unclassified was decreased under the treatment of probiotics. The serum metabolome results showed significant changes in serum metabolites during the establishment of the GIOP rat model, as well as treatment with alendronate and probiotics. Glycerophospholipid metabolism, Propanoate metabolism, Glutathione metabolism and Arachidonic acid metabolism were identified as the main altered metabolic pathways during osteoporosis modeling. Pentose and glucuronate interconversions and Glycerophospholipid metabolism seem the main altered metabolic pathways after the application of *L. plantarum*.

Recently, animal experiments from rats and mice have confirmed probiotics’ mitigating and therapeutic effects on osteoporosis. Leena et al. showed that the use of *Lactobacillus rhamnosus* gavage could inhibit bone resorption and regulate the differentiation of osteoblasts and osteoclasts in osteoporotic rats, ultimately reducing bone loss and restoring bone cortical and trabecular content (59). Similarly, another study showed that supplementation with *Bifidobacterium longum* inhibited osteoclast differentiation and activity, further improving bone microarchitecture, bone volume and bone strength in OP mice (60). In addition, a variety of probiotics including *Prevotella histicola*, *Lactobacillus paracasei*, *Lactobacillus reuteri*, *Probiotic Prevotella histicola*, *Bacillus subtilis*, and *Propionibacterium freudenreichii* are beneficial for improving bone loss and alleviating osteoporosis, and show favorable therapeutic effects (61–66). *L. plantarum*, a genus of lactic acid bacteria, is regarded as one of the most desirable probiotic species due to its high tolerance to gastrointestinal conditions, altering the intestinal microbiota, decreasing the abundance of harmful bacteria, increasing the abundance of beneficial bacteria, and inhibiting the propagation of pathogens.

As a result of dramatic advances in medical molecular imaging, computed tomography scanners are not limited to human use and have been developed for use in many animals. Currently, the Micro-CT Skyscan 1276 system is widely used in bone disease studies due to its high stability and resolution in analyzing bone structures. In addition to 3D and 2D microstructural mapping of bone, stereoscopic scanning of bone structure calculates a variety of quantitative data used to measure bone status, such as BMD, Intersection surface, Bone surface density and Tb.Th. At the IOF World Congress on Osteoporosis, BMD was recognized as one of the predictors of fracture and an assessment tool for the level of recovery from osteoporosis (67). It has been shown that every 10% decrease in hip bone BMD leads to a 2.5-fold increase in fracture risk (68). The quantity, thickness and density of trabecular bone are closely related to cancellous and cortical bone strength. A recent study reported by Shen-Shih Chiang et al. showed that supplementation with *Lactobacillus paracasei* significantly alleviated ovariectomy-induced postmenopausal osteoporosis in rats (34). Micro-CT showed that BMD, Tb.Th and Tb.N increased after probiotic intervention and decreased Tb.Sp. This is similar to our findings that all parameters of the femur of GIOP rats changed for the better after treatment with *L. plantarum*. The trend and magnitude of this change was comparable to that of the anti-osteoporotic drug alendronate.

Rat gut microbiota composition was significantly altered in the construction of the GIOP model and the subsequent treatment with *L. plantarum* and alendronate. According to previous studies osteoporosis causes a decrease in gut microbial diversity, which recovers when properly treated. This is consistent with our study. The alpha diversity of the intestinal microbiota was significantly increased in the DEX-LP and DEX-ALN groups of rats (including ACE Index, Chao1 Index, Shannon Index and PD-whole-tree Index). This suggests that a greater diversity of bacteria was observed in the treatment-imposed GIOP rats, which contributed to increased intestinal resistance. Increased abundance of Proteobacteria in GIOP rats leads to dysbiosis of intestinal flora and increased risk of disease. In addition, another study found that Bacteroidota can reduce bone resorption, increase bone formation and protect bones by promoting osteoblast differentiation and inhibiting osteoclast formation (69, 70). The Firmicutes/Bacteroidetes ratio was restored after *L. plantarum* and alendronate intervention. Elevated or decreased F/B ratios have been associated with the development of obesity or intestinal disorders, respectively (71). The use of appropriate probiotics can produce different effects. For example, treating probiotics like *S. boulardii*, *L. salivarius*, *L. sakei* and *L. rhamnosus* can reduce the F/B ratio and body weight. In addition, the administration of probiotics like B. lactis, L. acidophilus, *L. fermentum*, *L. reuteri* and *L. plantarum* increased the F/B ratio and associated with enteroprotective effects and immunosuppression. A study by Yong Ma et al. on metabolic disorders and high-fat diets showed that treatment with *L. plantarum* significantly decreased the F/B ratio (72), which is the same as our experimental findings. In addition, Yuan-Wei Zhang et al. showed that the use of *Prevotella histicola* in the treatment of osteoporosis also decreased F/B. The decrease in F/B values caused by probiotics or anti-osteoporotic drug therapy may be related to a decrease in inflammation and oxidative stress caused by altered gut microbial abundance.

Application of *L. plantarum* increased the abundance of the beneficial bacteria *Lachnospiraceae*_NK4A136_group, *Ruminococcus*, UCG_005, *Oscillospiraceae* unclassified, *Romboutsia* and *Christensenellaceae*_R_7_group, and decreases the abundance of the harmful bacterium *Desulfovibrionaceae* unclassified. The trend is similar to GIOP therapeutic drug alendronate. Among them, *Lachnospiraceae*_NK4A136_group, *Ruminococcus*, *Romboutsia*, *Christensenellaceae*_R_7_group can regulate osteoporosis by increasing the production of Short-chain fatty acids (SCFA), which include propionic, acetic and butyric acids (73, 74). SCFA is a major metabolite produced by prebiotics (dietary fiber) via microbial fermentation in the gut. A small portion of it escapes absorption by the colon and is transported into the portal circulation (75). Sébastien Lucas et al. demonstrated that SCFA inhibits osteoclast differentiation and thus protects bone mass by down-regulating osteoclast activation-associated genes such as TRAF6 and NFATc1, leading to decreased oxidative phosphorylation and enhanced glycolysis (70). TRAF6 binding to RANK controls the formation, activation and survival of OC through multiple signaling pathways, including ERK/JNK/MAPK signaling pathway, NFATc1 signaling pathway, Ca^2+^ signaling pathway and PI3K/Akt/GSK3β signaling pathway (76). NFATc1, as a major regulator of OC, plays an essential role in OC differentiation and maturation, promoting expression of multiple bone resorption-associated genes and proteins, including Ctsk, c-Fos, Acp5 and Atp6vod2 (77). Moreover, the formation of SCFAs is accompanied by fermentation and hydrolysis reactions, and the pH of the intestinal luminal contents decreases (78). Elevated H^+^ levels in the colon hinder the formation of complexes between calcium and negatively charged metabolites such as phytates and oxalates. Consequently, the higher concentration of retained calcium enhances mineral absorption, leading to subsequent bone mineralization.

In addition, there is no direct evidence that osteoporosis is associated with *UCG_005*, *Oscillospiraceae unclassified* and *Desulfovibrionaceae*. However, numerous studies have shown that gut microbes can regulate bone remodeling by influencing the body’s inflammatory state. Certain bacteria influence bone metabolism by mediating inflammation. *Oscillospiraceae* unclassified was negatively correlated with cellular inflammatory factors (IL-6, TNF-α, MCP-1) (79); *UCG-005* was inversely associated with the expression of pro-inflammatory cytokines (IL-12, IL-11, IL-8, IL-6, TNF-α, IL-1β and MCP-1) (80–82); *Lachnospiraceae*_NK4A136_group was inversely associated with the expression of cellular inflammatory factors (IL-1β, TNF-α, PGE-2, IL-6, and IFN-γ) (83–85); *Desulfovibrionaceae unclassified* was positively correlated with the expression of cellular inflammatory factors (TNF-α, IL-6, IL-1β, and MCP-1) (86, 87). A large increase in pro-inflammatory cytokines can act directly or indirectly on osteoblasts or osteoclasts, promoting the differentiation of monocytes into mature osteoclasts, increased bone resorption, and decreased bone mass. Studies have shown that several pro-inflammatory cytokines have pro- and/or anti-osteoclastogenic properties and can target osteoclasts either directly or through the nuclear factor-κB receptor activator (RANK)/RANK ligand (RANKL)/osteoclastogenic protein (OPG) system. TNF-α mediates RANKL expression and osteoblast apoptosis through the NF-κB and PI3K/Akt pathways. TNF-α synergistically induces RANKL-induced proliferation and differentiation of osteoclasts and inhibits their apoptosis (88). The production of IFN-γ by T cells exerts a potent acceleration on the JAK-STAT1 signaling pathway, leading to the rapid degradation of TRAF6. This process subsequently disrupts the RANKL-RANK signaling cascade, ultimately resulting in the suppression of osteoclastogenesis (89).

Serum metabolites provide a reflection of changes in endogenous metabolite-associated metabolic pathways in systems, organs, or the organism. We used serum metabolomics studies in order to assess the efficacy of *L. plantarum* in the treatment of GIOP rats and its intrinsic mechanisms. Differential metabolites in GIOP rats after treatment with *L. plantarum* included Pyrazine, Tetrahydrocorticosterone, 6-Hydroxymelatonin, LysoPE (24:6 (6Z,9Z,12Z,15Z,18Z,21Z)/0. 0), gamma-Glutamylcysteine, Glutamylphenylalanine, dolichyl D-xylosyl phosphates, Yohimbine, Merodesmosine, 3b,15b,17a-Trihydroxy- pregnenone and 2,2-Dichloro-1,1-ethanediol, etc. Compared to the DEX group, the DEX-LP group exhibited notably elevated pyrazine levels, contributing to preserving bone mass and ameliorating GIOP. Glucocorticoids are known to decrease the activity of bone marrow mesenchymal stem cells (BMSCs) and increase the growth and differentiation of osteoclasts. Pyrazine was shown to increase the expression of the osteogenic genes OSX, osteocalcin, collagen type I α1, and ALP and decreased the expression of the osteogenesis-related genes cathepsin K and TRAP (90). This results in osteoblast-mediated bone formation and reduced osteoclast-mediated bone resorption. Moreover, pyrazine induces autophagy in a manner dependent on the AMP-activated protein kinase (AMPK) and mammalian target of rapamycin (mTOR) pathway. This process serves to avert apoptosis in BMSCs (91). Ultimately, maintaining bone mass ameliorates the adverse effects of glucocorticoids. Increased serum levels of gamma-Glutamylcysteine (gamma-Glu) were associated with BMSC upregulation (92). Our study showed that *L. plantarum* significantly increased gamma-Glu levels. In addition, Hiroyuki Kanzaki et al. showed that decreased levels of gamma-Glu, a cytoprotective enzyme, correlated with osteoclast precursor proliferation and differentiation by mediating the up-regulation of Keap1, which is a negative regulator of Nrf2 (92). Indoxyl sulfate was significantly increased in serum after *L. plantarum* treatment. Numerous studies have shown a significant relationship between the development and treatment of osteoporosis and Glycerophospholipid metabolism (93–98). This is consistent with our findings. Analysis of potential signaling pathways involved in osteoporosis by differential metabolites revealed that the CON vs DEX group and DEX vs DEX-LP group of differential microorganisms were significantly enriched in Glycerophospholipid metabolism.

In this investigation, *L. plantarum* demonstrated a protective effect against GIOP in rats, assessed through analysis of both the gut microbiome and serum metabolome. Moreover, existing studies have consistently highlighted the preventive and therapeutic efficacy of *L. plantarum* in animal models of primary osteoporosis, encompassing ovariectomized models simulating postmenopausal women with estrogen deficiency-induced osteoporosis and orchiectomized models representing elderly men with bone loss due to reduced sex hormone levels (99–102). Similar to our study, Micro-CT results showed that *L. plantarum* treatment significantly increased BMD, Tb.N, trabecular volume and Tb.Th, and decreased Tb.Sp in ovariectomized rats (101). Additionally, *L. plantarum* treatment upregulated mRNA expression of the tight junction proteins ZO-1 and occludin in enterocytes, promoting the integrity of the intestinal barrier (101, 102). Concomitantly, there was a reduction in the expression of pro-inflammatory cytokines TNF-α, IL-1β, and IL-8, indicating a mitigated inflammatory response in the organism (102). This protective effect may be linked to the interaction of *L. plantarum* with RANKL and RANK, triggering the expression of c-Fos/Nfatc1 to regulate MAPK/AP-1 signaling and Nfatc1 transcription. Consequently, this interaction inhibits osteoclast differentiation and bone resorption (40, 42). In orchiectomized mice, *L. plantarum* exerts therapeutic effects by elevating short-chain fatty acid (SCFA) levels in the cecum and influencing the gut flora structure (99).

## 5 Conclusion

In this study, we used the glucocorticoid dexamethasone to induce rats suffering from osteoporosis and explored its therapeutic potential by using the probiotic *L. plantarum* by gavage and made a reference to alendronate, a commercially circulating GIOP therapeutic drug. The study showed that like alendronate, *L. plantarum* significantly alleviated osteoporosis in rats. Micro-CT results showed that the probiotic treatment restored various parameters of bone tissue microstructure such as BMD, Tb.Th, and Tb.N. Sequencing of rat colon contents revealed that *L. plantarum* treatment increased the α-diversity of gut microorganisms. The abundance of beneficial bacteria exhibited an increase, concomitant with a decrease in the abundance of harmful bacteria. Serum metabolome results showed significant changes in serum metabolites in all groups. Differential metabolites were significantly enriched in Glycerophospholipid metabolism both during osteoporosis modeling and *L. plantarum* treatment. Probiotics significantly increased serum levels of Pyrazine and gamma-Glutamylcysteine, which both inhibited osteoclast differentiation and induced osteoblast differentiation, thereby maintaining bone mass. These findings suggest that *L. plantarum* holds promise as a novel treatment strategy for GIOP, offering a potential alternative to current medications with their associated adverse effects. Additional research is warranted to comprehensively elucidate the underlying mechanisms and optimize the dosage and duration of *L. plantarum* treatment. This optimization will contribute to realizing the utmost therapeutic benefits in managing GIOP.

## Data availability statement

The datasets presented in this study can be found in online repositories. The names of the repository/repositories and accession number(s) can be found below: https://www.ncbi.nlm.nih.gov/genbank/, PRJNA924014.

## Ethics statement

The animal study was approved by Animal Care Committee of Hunan Agricultural University. The study was conducted in accordance with the local legislation and institutional requirements.

## Author contributions

SL: Data curation, Writing – original draft, Writing – review & editing, Investigation. XH: Data curation, Writing – review & editing. NL: Writing – review & editing, Data curation. JC: Writing – review & editing, Funding acquisition. GL: Conceptualization, Methodology, Writing – review & editing, Supervision. SH: Funding acquisition, Supervision, Writing – review & editing.

## References

[1] Föger-Samwald U Dovjak P Azizi-Semrad U Kerschan-Schindl K Pietschmann P. Osteoporosis: Pathophysiology and therapeutic options. Excli J (2020) 19:1017–37. doi: 10.17179/excli2020-2591 PMC7415937 32788914

[2] Anam AK Insogna K. Update on osteoporosis screening and management. Med Clin North Am (2021) 105:1117–34. doi: 10.1016/j.mcna.2021.05.016 34688418

[3] Rozenberg S Bruyère O Bergmann P Cavalier E Gielen E Goemaere S. How to manage osteoporosis before the age of 50. Maturitas (2020) 138:14–25. doi: 10.1016/j.maturitas.2020.05.004 32631584

[4] Zijlstra GJ Ten Hacken NH Hoffmann RF van Oosterhout AJ Heijink IH. Interleukin-17A induces glucocorticoid insensitivity in human bronchial epithelial cells. Eur Respir J (2012) 39:439–45. doi: 10.1183/09031936.00017911 21828034

[5] Zuckner J Uddin J Ramsey RH. INTRAMUSCULAR ADMINISTRATION OF STEROIDS IN TREATMENT OF RHEUMATOID ARTHRITIS. Ann Rheum Dis (1964) 23:456–62. doi: 10.1136/ard.23.6.456 PMC1030961 14229579

[6] Zheng H Bu S. Dexamethasone and number of days alive without life support in adults with COVID-19 and severe hypoxemia. Jama (2022) 327:682. doi: 10.1001/jama.2021.24522 35166810

[7] Zhu L Wang Z Lu X Xiao Z Zhou Y Wang L. Successful management and treatment of SLE-associated hypophysitis. Immunol Res (2020) 68:107–9. doi: 10.1007/s12026-020-09118-8 32152821

[8] Zielińska KA Van Moortel L Opdenakker G De Bosscher K Van den Steen PE. Endothelial response to glucocorticoids in inflammatory diseases. Front Immunol (2016) 7:592. doi: 10.3389/fimmu.2016.00592 28018358 PMC5155119

[9] Sousa LH Moura EV Queiroz AL Val D Chaves H Lisboa M. Effects of glucocorticoid-induced osteoporosis on bone tissue of rats with experimental periodontitis. Arch Oral Biol (2017) 77:55–61. doi: 10.1016/j.archoralbio.2017.01.014 28178585

[10] Ma Y Yang H Huang J. Icariin ameliorates dexamethasone−induced bone deterioration in an experimental mouse model via activation of microRNA−186 inhibition of cathepsin K. Mol Med Rep (2018) 17:1633–41. doi: 10.3892/mmr.2017.8065 PMC5780104 29257214

[11] den Uyl D Bultink IE Lems WF. Advances in glucocorticoid-induced osteoporosis. Curr Rheumatol Rep (2011) 13:233–40. doi: 10.1007/s11926-011-0173-y PMC3092927 21365209

[12] Kim HJ Zhao H Kitaura H Bhattacharyya S Brewer JA Muglia LJ. Glucocorticoids suppress bone formation via the osteoclast. J Clin Invest (2006) 116:2152–60. doi: 10.1172/JCI28084 PMC1518793 16878176

[13] Arafa EA Elgendy NO Elhemely MA Abdelaleem EA Mohamed WR. Diosmin mitigates dexamethasone-induced osteoporosis in *vivo*: Role of Runx2, RANKL/OPG, and oxidative stress. BioMed Pharmacother (2023) 161:114461. doi: 10.1016/j.biopha.2023.114461 36889109

[14] Hu H Li Z Lu M Yun X Li W Liu C. Osteoactivin inhibits dexamethasone-induced osteoporosis through up-regulating integrin β1 and activate ERK pathway. BioMed Pharmacother (2018) 105:66–72. doi: 10.1016/j.biopha.2018.05.051 29843046

[15] Li J Zhang N Huang X Xu J Fernandes JC Dai K. Dexamethasone shifts bone marrow stromal cells from osteoblasts to adipocytes by C/EBPalpha promoter methylation. Cell Death Dis (2013) 4:e832. doi: 10.1038/cddis.2013.348 24091675 PMC3824658

[16] Zhang JY Zhong YH Chen LM Zhuo XL Zhao LJ Wang YT. Recent advance of small-molecule drugs for clinical treatment of osteoporosis: A review. Eur J Med Chem (2023) 259:115654. doi: 10.1016/j.ejmech.2023.115654 37467618

[17] de Villiers TJ Gass ML Haines CJ Hall JE Lobo RA Pierroz DD. Global consensus statement on menopausal hormone therapy. Climacteric (2013) 16:203–4. doi: 10.3109/13697137.2013.771520 23488524

[18] Komm BS Mirkin S. An overview of current and emerging SERMs. J Steroid Biochem Mol Biol (2014) 143:207–22. doi: 10.1016/j.jsbmb.2014.03.003 24667357

[19] Davison S Davis SR. Hormone replacement therapy: current controversies. Clin Endocrinol (Oxf) (2003) 58:249–61. doi: 10.1046/j.1365-2265.2003.01774.x 12608928

[20] Fleisch HA. Bisphosphonates: preclinical aspects and use in osteoporosis. Ann Med (1997) 29:55–62. doi: 10.3109/07853899708998743 9073324

[21] Oyhanart SR Escudero ND Mandalunis PM. Effect of alendronate on the mandible and long bones: an experimental study in vivo. Pediatr Res (2015) 78:618–25. doi: 10.1038/pr.2015.163 26331769

[22] Miller PD Jamal SA Evenepoel P Eastell R Boonen S. Renal safety in patients treated with bisphosphonates for osteoporosis: a review. J Bone Miner Res (2013) 28:2049–59. doi: 10.1002/jbmr.2058 23907861

[23] Drake MT Clarke BL Khosla S. Bisphosphonates: mechanism of action and role in clinical practice. Mayo Clin Proc (2008) 83:1032–45. doi: 10.4065/83.9.1032 PMC2667901 18775204

[24] Russell RG. Bisphosphonates: the first 40 years. Bone (2011) 49:2–19. doi: 10.1016/j.bone.2011.04.022 21555003

[25] Tonino RP Meunier PJ Emkey R Rodriguez-Portales JA Menkes CJ Wasnich RD. Skeletal benefits of alendronate: 7-year treatment of postmenopausal osteoporotic women. Phase III Osteoporosis Treatment Study Group. J Clin Endocrinol Metab (2000) 85:3109–15. doi: 10.1210/jcem.85.9.6777 10999794

[26] Liberman UA Weiss SR Bröll J Minne HW Quan H Bell NH. Effect of oral alendronate on bone mineral density and the incidence of fractures in postmenopausal osteoporosis. The Alendronate Phase III Osteoporosis Treatment Study Group. N Engl J Med (1995) 333:1437–43. doi: 10.1056/NEJM199511303332201 7477143

[27] Kavanagh KL Guo K Dunford JE Wu X Knapp S Ebetino FH. The molecular mechanism of nitrogen-containing bisphosphonates as antiosteoporosis drugs. Proc Natl Acad Sci USA (2006) 103:7829–34. doi: 10.1073/pnas.0601643103 PMC1472530 16684881

[28] Benford HL McGowan NW Helfrich MH Nuttall ME Rogers MJ. Visualization of bisphosphonate-induced caspase-3 activity in apoptotic osteoclasts in vitro. Bone (2001) 28:465–73. doi: 10.1016/s8756-3282(01)00412-4 11344045

[29] Stapleton M Sawamoto K Alméciga-Díaz CJ Mackenzie WG Mason RW Orii T. Development of bone targeting drugs. Int J Mol Sci (2017) 18(7):1345. doi: 10.3390/ijms18071345 28644392 PMC5535838

[30] Zaiss MM Jones RM Schett G Pacifici R. The gut-bone axis: how bacterial metabolites bridge the distance. J Clin Invest (2019) 129:3018–28. doi: 10.1172/JCI128521 PMC6668676 31305265

[31] Yan J Takakura A Zandi-Nejad K Charles JF. Mechanisms of gut microbiota-mediated bone remodeling. Gut Microbes (2018) 9:84–92. doi: 10.1080/19490976.2017.1371893 28961041 PMC5914914

[32] Villa CR Ward WE Comelli EM. Gut microbiota-bone axis. Crit Rev Food Sci Nutr (2017) 57:1664–72. doi: 10.1080/10408398.2015.1010034 26462599

[33] Zhou B Yuan Y Zhang S Guo C Li X Li G. Intestinal flora and disease mutually shape the regional immune system in the intestinal tract. Front Immunol (2020) 11:575. doi: 10.3389/fimmu.2020.00575 32318067 PMC7147503

[34] Chiang SS Pan TM. Antiosteoporotic effects of Lactobacillus -fermented soy skim milk on bone mineral density and the microstructure of femoral bone in ovariectomized mice. J Agric Food Chem (2011) 59:7734–42. doi: 10.1021/jf2013716 21668014

[35] McCabe LR Irwin R Schaefer L Britton RA. Probiotic use decreases intestinal inflammation and increases bone density in healthy male but not female mice. J Cell Physiol (2013) 228:1793–8. doi: 10.1002/jcp.24340 PMC4091780 23389860

[36] Zhu Y Wang X Pan W Shen X He Y Yin H. Exopolysaccharides produced by yogurt-texture improving Lactobacillus plantarum RS20D and the immunoregulatory activity. Int J Biol Macromol (2019) 121:342–9. doi: 10.1016/j.ijbiomac.2018.09.201 30287381

[37] Park KB Oh SH. Cloning, sequencing and expression of a novel glutamate decarboxylase gene from a newly isolated lactic acid bacterium, Lactobacillus brevis OPK-3. Bioresour Technol (2007) 98:312–9. doi: 10.1016/j.biortech.2006.01.004 16500100

[38] Zhou M Zheng X Zhu H Li L Zhang L Liu M. Effect of Lactobacillus plantarum enriched with organic/inorganic selenium on the quality and microbial communities of fermented pickles. Food Chem (2021) 365:130495. doi: 10.1016/j.foodchem.2021.130495 34243128

[39] Morato-Martínez M López-Plaza B Santurino C Palma-Milla S Gómez-Candela C. A dairy product to reconstitute enriched with bioactive nutrients stops bone loss in high-risk menopausal women without pharmacological treatment. Nutrients (2020) 12(8):2203. doi: 10.3390/nu12082203 32722015 PMC7468696

[40] Yang LC Lin SW Li IC Chen YP Tzu SY Chou W. Lactobacillus plantarum GKM3 and Lactobacillus paracasei GKS6 Supplementation Ameliorates Bone Loss in Ovariectomized Mice by Promoting Osteoblast Differentiation and Inhibiting Osteoclast Formation. Nutrients (2020) 12(7):1914. doi: 10.3390/nu12071914 32605314 PMC7401263

[41] Tsai WH Lin WC Chou CH Yang LC. The probiotic Lactiplantibacillus plantarum attenuates ovariectomy-induced osteoporosis through osteoimmunological signaling. Food Funct (2023) 14:6929–40. doi: 10.1039/D3FO00681F 37431637

[42] Myeong JY Jung HY Chae HS Cho HH Kim DK Jang YJ. Protective effects of the postbiotic lactobacillus plantarum MD35 on bone loss in an ovariectomized mice model. Probiotics Antimicrob Proteins (2023). doi: 10.1007/s12602-023-10065-7 PMC10987357 37002419

[43] Lee CS Kim JY Kim BK Lee IO Park NH Kim SH. Lactobacillus-fermented milk products attenuate bone loss in an experimental rat model of ovariectomy-induced post-menopausal primary osteoporosis. J Appl Microbiol (2021) 130:2041–62. doi: 10.1111/jam.14852 32920885

[44] Wang H Yang L Chao J. Antiosteoporosis and bone protective effect of dieckol against glucocorticoid-induced osteoporosis in rats. Front Endocrinol (Lausanne) (2022) 13:932488. doi: 10.3389/fendo.2022.932488 36060953 PMC9437630

[45] Malkawi AK Alzoubi KH Jacob M Matic G Ali A Al Faraj A. Metabolomics based profiling of dexamethasone side effects in rats. Front Pharmacol (2018) 9:46. doi: 10.3389/fphar.2018.00046 29503615 PMC5820529

[46] Yang Y Yu T Tang H Ren Z Li Q Jia J. Ganoderma lucidum Immune Modulator Protein rLZ-8 Could Prevent and Reverse Bone Loss in Glucocorticoids-Induced Osteoporosis Rat Model. Front Pharmacol (2020) 11:731. doi: 10.3389/fphar.2020.00731 32508652 PMC7248554

[47] Yang X Jiang T Wang Y Guo L. The role and mechanism of SIRT1 in resveratrol-regulated osteoblast autophagy in osteoporosis rats. Sci Rep (2019) 9:18424. doi: 10.1038/s41598-019-44766-3 31804494 PMC6895060

[48] Ding S Ma Y Liu G Yan W Jiang H Fang J. Lactobacillus brevis alleviates DSS-induced colitis by reprograming intestinal microbiota and influencing serum metabolome in murine model. Front Physiol (2019) 10:1152. doi: 10.3389/fphys.2019.01152 31620010 PMC6759783

[49] Zullo AR Zhang T Beaudoin FL Lee Y McConeghy KW Kiel DP. Pain treatments after hip fracture among older nursing home residents. J Am Med Dir Assoc (2018) 19:174–6. doi: 10.1016/j.jamda.2017.11.008 PMC5803325 29287695

[50] Zuo H Zheng T Wu K Yang T Wang L Nima Q. High-altitude exposure decreases bone mineral density and its relationship with gut microbiota: Results from the China multi-ethnic cohort (CMEC) study. Environ Res (2022) 215:114206. doi: 10.1016/j.envres.2022.114206 36058270

[51] Zura R Xiong Z Einhorn T Watson JT Ostrum RF Prayson MJ. Epidemiology of fracture nonunion in 18 human bones. JAMA Surg (2016) 151:e162775. doi: 10.1001/jamasurg.2016.2775 27603155

[52] Weinstein RS. Clinical practice. Glucocorticoid-induced bone disease. N Engl J Med (2011) 365:62–70. doi: 10.1056/NEJMcp1012926 21732837

[53] Weare-Regales N Hudey SN Lockey RF. Practical guidance for prevention and management of glucocorticoid-induced osteoporosis for the allergist/immunologist. J Allergy Clin Immunol Pract (2021) 9:1841–50. doi: 10.1016/j.jaip.2020.12.050 33444813

[54] Weinstein RS. Glucocorticoid-induced osteoporosis. Rev Endocr Metab Disord (2001) 2:65–73. doi: 10.1023/A:1010007108155 11708295

[55] Zhang YW Cao MM Li YJ Dai GC Lu PP Zhang M. The regulative effect and repercussion of probiotics and prebiotics on osteoporosis: involvement of brain-gut-bone axis. Crit Rev Food Sci Nutr (2022) 63(25):7510–28. doi: 10.1080/10408398.2022.2047005 35234534

[56] Zhang YW Li YJ Lu PP Dai GC Chen XX Rui YF. The modulatory effect and implication of gut microbiota on osteoporosis: from the perspective of “brain-gut-bone” axis. Food Funct (2021) 12:5703–18. doi: 10.1039/D0FO03468A 34048514

[57] Liu J Liu J Liu L Zhang G Peng X. Reprogrammed intestinal functions in Astragalus polysaccharide-alleviated osteoporosis: combined analysis of transcriptomics and DNA methylomics demonstrates the significance of the gut-bone axis in treating osteoporosis. Food Funct (2021) 12:4458–70. doi: 10.1039/D1FO00113B 33881125

[58] Li B Liu M Wang Y Gong S Yao W Li W. Puerarin improves the bone micro-environment to inhibit OVX-induced osteoporosis via modulating SCFAs released by the gut microbiota and repairing intestinal mucosal integrity. BioMed Pharmacother (2020) 132:110923. doi: 10.1016/j.biopha.2020.110923 33125971

[59] Sapra L Dar HY Bhardwaj A Pandey A Kumari S Azam Z. Lactobacillus rhamnosus attenuates bone loss and maintains bone health by skewing Treg-Th17 cell balance in Ovx mice. Sci Rep (2021) 11:1807. doi: 10.1038/s41598-020-80536-2 33469043 PMC7815799

[60] Sapra L Shokeen N Porwal K Saini C Bhardwaj A Mathew M. Bifidobacterium longum Ameliorates Ovariectomy-Induced Bone Loss via Enhancing Anti-Osteoclastogenic and Immunomodulatory Potential of Regulatory B Cells (Bregs). Front Immunol (2022) 13:875788. doi: 10.3389/fimmu.2022.875788 35693779 PMC9174515

[61] Wang Z Chen K Wu C Chen J Pan H Liu Y. An emerging role of Prevotella histicola on estrogen deficiency-induced bone loss through the gut microbiota-bone axis in postmenopausal women and in ovariectomized mice. Am J Clin Nutr (2021) 114:1304–13. doi: 10.1093/ajcn/nqab194 34113963

[62] Liu TH Tsai TY Pan TM. Effects of an ethanol extract from Lactobacillus paracasei subsp. paracasei NTU 101 fermented skimmed milk on lipopolysaccharide-induced periodontal inflammation in rats. Food Funct (2018) 9:4916–25. doi: 10.1039/C8FO01303A 30178812

[63] Nilsson AG Sundh D Bäckhed F Lorentzon M. Lactobacillus reuteri reduces bone loss in older women with low bone mineral density: a randomized, placebo-controlled, double-blind, clinical trial. J Intern Med (2018) 284:307–17. doi: 10.1111/joim.12805 29926979

[64] Zhang YW Cao MM Li YJ Sheng RW Zhang RL Wu MT. The preventive effects of probiotic prevotella histicola on the bone loss of mice with ovariectomy-mediated osteoporosis. Microorganisms (2023) 11(4):950. doi: 10.3390/microorganisms11040950 37110373 PMC10146713

[65] Sojan JM Licini C Marcheggiani F Carnevali O Tiano L Mattioli-Belmonte M. Bacillus subtilis modulated the expression of osteogenic markers in a human osteoblast cell line. Cells (2023) 12(3):364. doi: 10.3390/cells12030364 36766709 PMC9913848

[66] Yeom J Ma S Lim YH. Probiotic propionibacterium freudenreichii MJ2 enhances osteoblast differentiation and mineralization by increasing the OPG/RANKL ratio. Microorganisms (2021) 9(4):673. doi: 10.3390/microorganisms9040673 33805153 PMC8064112

[67] Häntzschel H. IOF world congress on osteoporosis 14th - 18th may 2004, rio de janeiro. Z Rheumatol (2004) 63:501–3. doi: 10.1007/s00393-004-0660-x 15605218

[68] Klotzbuecher CM Ross PD Landsman PB Abbott TA 3rd Berger M. Patients with prior fractures have an increased risk of future fractures: a summary of the literature and statistical synthesis. J Bone Miner Res (2000) 15:721–39. doi: 10.1359/jbmr.2000.15.4.721 10780864

[69] Chen TH Chen WM Hsu KH Kuo CD Hung SC. Sodium butyrate activates ERK to regulate differentiation of mesenchymal stem cells. Biochem Biophys Res Commun (2007) 355:913–8. doi: 10.1016/j.bbrc.2007.02.057 17331472

[70] Lucas S Omata Y Hofmann J Böttcher M Iljazovic A Sarter K. Short-chain fatty acids regulate systemic bone mass and protect from pathological bone loss. Nat Commun (2018) 9:55. doi: 10.1038/s41467-017-02490-4 29302038 PMC5754356

[71] Stojanov S Berlec A Štrukelj B. The influence of probiotics on the firmicutes/bacteroidetes ratio in the treatment of obesity and inflammatory bowel disease. Microorganisms (2020) 8(11):1715. doi: 10.3390/microorganisms8111715 33139627 PMC7692443

[72] Ma Y Fei Y Han X Liu G Fang J. Lactobacillus plantarum alleviates obesity by altering the composition of the gut microbiota in high-fat diet-fed mice. Front Nutr (2022) 9:947367. doi: 10.3389/fnut.2022.947367 35845812 PMC9280677

[73] Zhan Z Tang H Zhang Y Huang X Xu M. Potential of gut-derived short-chain fatty acids to control enteric pathogens. Front Microbiol (2022) 13:976406. doi: 10.3389/fmicb.2022.976406 36204607 PMC9530198

[74] Ye X Liu Y Hu J Gao Y Ma Y Wen D. Chlorogenic acid-induced gut microbiota improves metabolic endotoxemia. Front Endocrinol (Lausanne) (2021) 12:762691. doi: 10.3389/fendo.2021.762691 34975748 PMC8716487

[75] Liu XF Shao JH Liao YT Wang LN Jia Y Dong PJ. Regulation of short-chain fatty acids in the immune system. Front Immunol (2023) 14:1186892. doi: 10.3389/fimmu.2023.1186892 37215145 PMC10196242

[76] Wang G Chen K Ma C Wang C Chen D He J. Roburic acid attenuates osteoclastogenesis and bone resorption by targeting RANKL-induced intracellular signaling pathways. J Cell Physiol (2022) 237:1790–803. doi: 10.1002/jcp.30642 34796915

[77] Ma C Mo L Wang Z Peng D Zhou C Niu W. Dihydrotanshinone I attenuates estrogen-deficiency bone loss through RANKL-stimulated NF-κB, ERK and NFATc1 signaling pathways. Int Immunopharmacol (2023) 123:110572. doi: 10.1016/j.intimp.2023.110572 37572501

[78] Scholz-Ahrens KE Schrezenmeir J. Inulin, oligofructose and mineral metabolism - experimental data and mechanism. Br J Nutr (2002) 87 Suppl 2:S179–86. doi: 10.1079/BJN/2002535 12088516

[79] Zhao L Zhang Q Ma W Tian F Shen H Zhou M. A combination of quercetin and resveratrol reduces obesity in high-fat diet-fed rats by modulation of gut microbiota. Food Funct (2017) 8:4644–56. doi: 10.1039/C7FO01383C 29152632

[80] Wang Z Liang Y Yu J Zhang D Ren L Zhang Z. Guchang Zhixie Wan protects mice against dextran sulfate sodium-induced colitis through modulating the gut microbiota in colon. J Ethnopharmacol (2020) 260:112991. doi: 10.1016/j.jep.2020.112991 32442592

[81] Yao ZY Li XH Zuo L Xiong Q He WT Li DX. Maternal sleep deprivation induces gut microbial dysbiosis and neuroinflammation in offspring rats. Zool Res (2022) 43:380–90. doi: 10.24272/j.issn.2095-8137.2022.023 PMC9113977 35362675

[82] Zhao H Gao X Liu Z Zhang L Fang X Sun J. Sodium alginate prevents non-alcoholic fatty liver disease by modulating the gut-liver axis in high-fat diet-fed rats. Nutrients (2022) 14(22):4846. doi: 10.3390/nu14224846 36432531 PMC9697635

[83] Zhou Q Qureshi N Xue B Xie Z Li P Gu Q. Preventive and therapeutic effect of Lactobacillus paracasei ZFM54 on Helicobacter pylori-induced gastritis by ameliorating inflammation and restoring gastric microbiota in mice model. Front Nutr (2022) 9:972569. doi: 10.3389/fnut.2022.972569 36091249 PMC9449542

[84] Zhou J Tang L Wang JS. Aflatoxin B1 induces gut-inflammation-associated fecal lipidome changes in F344 rats. Toxicol Sci (2021) 183:363–77. doi: 10.1093/toxsci/kfab096 34358323

[85] Zhen Y Ge L Xu Q Hu L Wei W Huang J. Normal light-dark and short-light cycles regulate intestinal inflammation, circulating short-chain fatty acids and gut microbiota in period2 gene knockout mice. Front Immunol (2022) 13:848248. doi: 10.3389/fimmu.2022.848248 35371053 PMC8971677

[86] Zhai Z Zhang F Cao R Ni X Xin Z Deng J. Cecropin A alleviates inflammation through modulating the gut microbiota of C57BL/6 mice with DSS-induced IBD. Front Microbiol (2019) 10:1595. doi: 10.3389/fmicb.2019.01595 31354682 PMC6635700

[87] Zhao N Ma Y Liang X Zhang Y Hong D Wang Y. Efficacy and mechanism of qianshan huoxue gao in acute coronary syndrome via regulation of intestinal flora and metabolites. Drug Des Devel Ther (2023) 17:579–95. doi: 10.2147/DDDT.S396649 PMC9968440 36855515

[88] Clowes JA Riggs BL Khosla S. The role of the immune system in the pathophysiology of osteoporosis. Immunol Rev (2005) 208:207–27. doi: 10.1111/j.0105-2896.2005.00334.x 16313351

[89] Zhou P Zheng T Zhao B. Cytokine-mediated immunomodulation of osteoclastogenesis. Bone (2022) 164:116540. doi: 10.1016/j.bone.2022.116540 36031187 PMC10657632

[90] Wang L Lu WG Shi J Zhang HY Xu XL Gao B. Anti−osteoporotic effects of tetramethylpyrazine via promoting osteogenic differentiation and inhibiting osteoclast formation. Mol Med Rep (2017) 16:8307–14. doi: 10.3892/mmr.2017.7610 28983593

[91] Wang L Zhang HY Gao B Shi J Huang Q Han YH. Tetramethylpyrazine protects against glucocorticoid-induced apoptosis by promoting autophagy in mesenchymal stem cells and improves bone mass in glucocorticoid-induced osteoporosis rats. Stem Cells Dev (2017) 26:419–30. doi: 10.1089/scd.2016.0233 27917698

[92] Kanzaki H Shinohara F Kajiya M Kodama T. The Keap1/Nrf2 protein axis plays a role in osteoclast differentiation by regulating intracellular reactive oxygen species signaling. J Biol Chem (2013) 288:23009–20. doi: 10.1074/jbc.M113.478545 PMC3743476 23801334

[93] Zhao H Li X Zhang D Chen H Chao Y Wu K. Integrative bone metabolomics-lipidomics strategy for pathological mechanism of postmenopausal osteoporosis mouse model. Sci Rep (2018) 8:16456. doi: 10.1038/s41598-018-34574-6 30405156 PMC6220250

[94] Wang J Yan D Zhao A Hou X Zheng X Chen P. Discovery of potential biomarkers for osteoporosis using LC-MS/MS metabolomic methods. Osteoporos Int (2019) 30:1491–9. doi: 10.1007/s00198-019-04892-0 30778642

[95] Partoazar A Goudarzi R. Phosphatidylserine liposomes containing curcumin inhibit bone loss in osteoporotic rats: A possible synergy through a common signaling pathway. J Food Biochem (2022) 46:e14120. doi: 10.1111/jfbc.14120 35229314

[96] Mao H Wang W Shi L Chen C Han C Zhao J. Metabolomics and physiological analysis of the effect of calcium supplements on reducing bone loss in ovariectomized rats by increasing estradiol levels. Nutr Metab (Lond) (2021) 18:76. doi: 10.1186/s12986-021-00602-y 34301294 PMC8305954

[97] Mangano KM Noel SE Lai CQ Christensen JJ Ordovas JM Dawson-Hughes B. Diet-derived fruit and vegetable metabolites show sex-specific inverse relationships to osteoporosis status. Bone (2021) 144:115780. doi: 10.1016/j.bone.2020.115780 33278656 PMC7856195

[98] Luo D Li J Chen K Rong X Guo J. Untargeted metabolomics reveals the protective effect of fufang zhenshu tiaozhi (FTZ) on aging-induced osteoporosis in mice. Front Pharmacol (2018) 9:1483. doi: 10.3389/fphar.2018.01483 30670964 PMC6331458

[99] Lawenius L Colldén H Horkeby K Wu J Grahnemo L Vandenput L. A probiotic mix partially protects against castration-induced bone loss in male mice. J Endocrinol (2022) 254:91–101. doi: 10.1530/JOE-21-0408 35661635 PMC9254303

[100] Chiang SS Liao JW Pan TM. Effect of bioactive compounds in lactobacilli-fermented soy skim milk on femoral bone microstructure of aging mice. J Sci Food Agric (2012) 92:328–35. doi: 10.1002/jsfa.4579 21815163

[101] Jin ES Kim JY Min J Jeon SR Choi KH Khan SA. Preliminary study on effect of lactiplantibacillus plantarum on osteoporosis in the ovariectomized rat. Food Sci Anim Resour (2023) 43:712–20. doi: 10.5851/kosfa.2023.e29 PMC10359845 37483997

[102] Lee CS Kim SH. Anti-inflammatory and Anti-osteoporotic Potential of Lactobacillus plantarum A41 and L. fermentum SRK414 as Probiotics. Probiotics Antimicrob Proteins (2020) 12:623–34. doi: 10.1007/s12602-019-09577-y 31372901

